# The association between COVID-19 vaccination and confirmed patients with hospitalization in Omicron era: A retrospective study

**DOI:** 10.1097/MD.0000000000036777

**Published:** 2023-12-29

**Authors:** Ming-Hung Chang, Kuang-Ming Liao

**Affiliations:** a Division of Chest Medicine, Department of Internal Medicine, Chi Mei Medical Center, Chiali, Tainan, Taiwan.

**Keywords:** COVID-19, Omicron variant, vaccination

## Abstract

With the emergence of Omicron variant of severe acute respiratory syndrome coronavirus 2, Taiwan has encountered the greatest coronavirus disease 2019 (COVID-19) pandemic since 2022 spring. We analyzed the characteristics, vaccinations, and outcomes of hospitalized COVID-19 patients quarantined in a dedicated ward. This retrospective study enrolled hospitalized COVID-19 patients in the dedicated wards of a district hospital in southern Taiwan from May 2022 to July 2022. We assessed in-hospital mortality, hospital length of stay (LOS), and dedicated ward LOS. Among 209 COVID-19 patients, the in-hospital mortality rates were 20.7% and 29.7% (*P* = .145) in patients with and without vaccination. A shorter dedicated ward LOS was noted in the vaccination group, with marginal statistical significance. Age, Charlson Comorbidity Index, and quick Sequential Organ Failure Assessment score were recognized as strong prognostic indicators for mortality in multivariable analysis. Vaccination demonstrated significant lower odds of death among relatively young populations in subgroup analysis. COVID-19 vaccination had significant efficacy in hospitalized COVID-19 patients in the relatively young group, and the effect may decline among individuals with advanced age and multiple comorbidities.

## 1. Introduction

Coronavirus disease 2019 (COVID-19), which designated by the World Health Organization as the name of the human disease caused by severe acute respiratory syndrome coronavirus 2 (SARS-CoV-2), was a novel ongoing disease from Wuhan, China. The disease pandemic already spread to over 200 countries and caused over 700 million confirmed cases of COVID-19, including over 6 million deaths. The mortality rate was about 1% and most of the cases were mild disease (not requiring hospitalization) or asymptomatic. Elders, racial disparities, and comorbidities such as diabetes, cardiovascular disease, chronic lung disease and obesity were established epidemiologic risk factors for severe illness and COVID-19 mortality.^[1,2]^

COVID-19 vaccination had been found to be efficacious at preventing symptomatic disease, reducing the risks of hospitalization and mortality.^[3–6]^ With the popularization of vaccine administration and herd immunity, the policy of Zero-COVID or FTTIS (Find, Test, Trace, Isolate and Support) might not be appropriate for post-pandemic era. Instead, many countries have begun to coexist with the virus. However the emergence of new and more transmissible variants of SARS-CoV-2 were detected, and the Omicron variant became the dominant variant of concern in 2022 worldwide.^[7]^ Taiwan has encountered the greatest COVID-19 pandemic since 2022 spring, causing over 10 million confirmed cases and over 18 thousand deaths. In this study, we analyzed the characteristics, vaccinations and outcomes of hospitalized COVID-19 patients quarantined in a dedicated ward.

## 2. Methods

### 2.1. Patient

This retrospective, single-center study enrolled hospitalized patients in dedicated wards of the district hospital in southern Taiwan, who were diagnosed with COVID-19 from May 2022 to July 2022. A positive nucleic acid amplification test (polymerase chain reaction) or rapid antigen test for SARS-CoV-2 confirmed the diagnosis of COVID-19.

The Charlson Comorbidity Index (CCI) was used to assess the baseline comorbidities, which contains myocardial infarction, congestive heart failure, peripheral vascular disease, cerebrovascular disease, dementia, chronic pulmonary disease, connective tissue disease, peptic ulcer disease, liver disease, diabetes mellitus, hemiplegia, chronic kidney disease, cancer and acquired immunodeficiency syndrome.^[8]^ Quick Sequential Organ Failure Assessment score (qSOFA) was calculated before admission to dedicated ward.^[9]^ Laboratory data included white blood cell count, absolute neutrophil count, absolute lymphocyte count, hemoglobin and platelet count.

### 2.2. Vaccination

Taiwan Food and Drug Administration approved ChAdOx1-S (Oxford/AstraZeneca), BNT162b2 (Pfizer–BioNTech), mRNA-1273 (Moderna), NVX-CoV2373 (Novavax), and MVC-COV1901 (Medigen) as COVID-19 vaccination, including primary series (ChAdOx1-S, BNT162b2, mRNA-1273, and MVC-COV1901), additional dose (BNT162b2, mRNA-1273, NVX-CoV2373, and MVC-COV1901) and booster dose (ChAdOx1-S, BNT162b2, mRNA-1273, NVX-CoV2373, and MVC-COV1901).

### 2.3. Specific therapies

The specific therapies for COVID-19 included anti-viral agents [nirmatrelvir + ritonavir (Paxlovid), molnupiravir (Lagevrio), and remdesivir (Veklury)] and corticosteroid (dexamethasone).^[10]^ Nirmatrelvir + ritonavir, molnupiravir and 3-day course of remdesivir were prescribed in patients without supplemental oxygen, who had risk factors for severe COVID-19. The comorbidities which Centers for Disease Control and Prevention classified as risk factors for severe COVID-19, included age ≥ 65 years, cancer, cerebrovascular disease, heart conditions (such as heart failure, coronary artery disease, or cardiomyopathies), chronic lung disease (limited to interstitial lung disease, pulmonary embolism, pulmonary hypertension, bronchiectasis, asthma and chronic obstructive pulmonary disease), tuberculosis, chronic liver disease (limited to cirrhosis, nonalcoholic fatty liver disease, alcoholic liver disease, autoimmune hepatitis), chronic kidney disease, diabetes mellitus, disabilities, dementia, mental health disorder, obesity (body mass index ≥ 30 kg/m^2^), immunodeficiency (such as human immunodeficiency virus, organ transplantation, use of corticosteroids or other immunosuppressive medications) and pregnancy.^[1]^

The indication of 5-day course of remdesivir was severe disease of COVID-19 (oxygen saturation ≤ 94% under room air or requirement of oxygen supplement) without endotracheal tube intubation. Dexamethasone was administered in severe disease with oxygen supplement, high flow nasal oxygen, noninvasive ventilation, intubation or extracorporeal membrane oxygenation.

### 2.4. Outcome measures

In this study, we assessed the in-hospital mortality rate, hospital length of stay (LOS) and dedicated ward LOS. In-hospital mortality rate referred to the percentage of COVID-19 patients who died from any cause while in the hospital including dedicated ward, ordinary ward or intensive care unit. Hospital LOS was calculated by subtracting day of admission or nosocomial infection from day of discharge or death. Dedicated ward LOS was defined as the duration of dedicated ward, and the patients who matched the criteria for release from isolation or passed away, would leave a dedicated ward.

The criteria for release from isolation published by Taiwan Centers for Disease Control, included (1) Severe disease: symptoms relief with one negative PCR test or cycle threshold value ≥ 30; one PCR cycle threshold value ≥ 27 after 15-day isolation. (2) Non-severe disease: symptom relief with 2 negative rapid antigen tests or one negative rapid antigen test over a 5-day period following the confirmed date; symptom relief above 7-day isolation without requirement of hospitalization.

### 2.5. Statistical analysis

All analyses were performed using the statistical software SPSS 24.0 (SPSS Inc., Chicago, IL). All categorical variables were analyzed by the Chi-squared test, except those with an expected frequency of <5, which were analyzed by Fisher exact test. Independent sample *t* test was used for mean comparisons of continuous variables between 2 groups. LOS curves were plotted using the Kaplan–Meier method and compared between groups using the log-rank test or generalized Wilcoxon test. Logistic regression, which calculated crude odd ratio and adjusted odds ratio (aOR), was used to assess the association between potential risk factors and the in-hospital mortality rate. All *P* values were based on a 2-tailed hypothesis, and statistical significance was assumed if *P* value < .05.

### 2.6. Ethics approval

This study was conducted ethically in accordance with the World Medical Association Declaration of Helsinki. It was approved by the Institutional Review Board of Chi Mei Medical Center, Taiwan (IRB No. 11111-J03) and was exempted from informed consent requirements owing to its retrospective design.

## 3. Results

### 3.1. Patients’ characteristics

**Figure 1.**
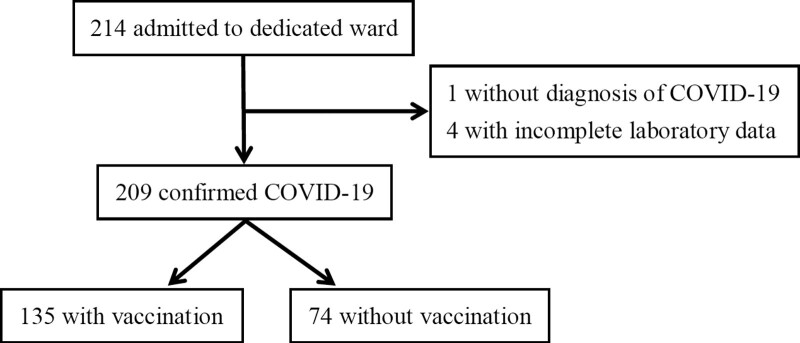
Flow chart of patients screening.

**Figure 2.**
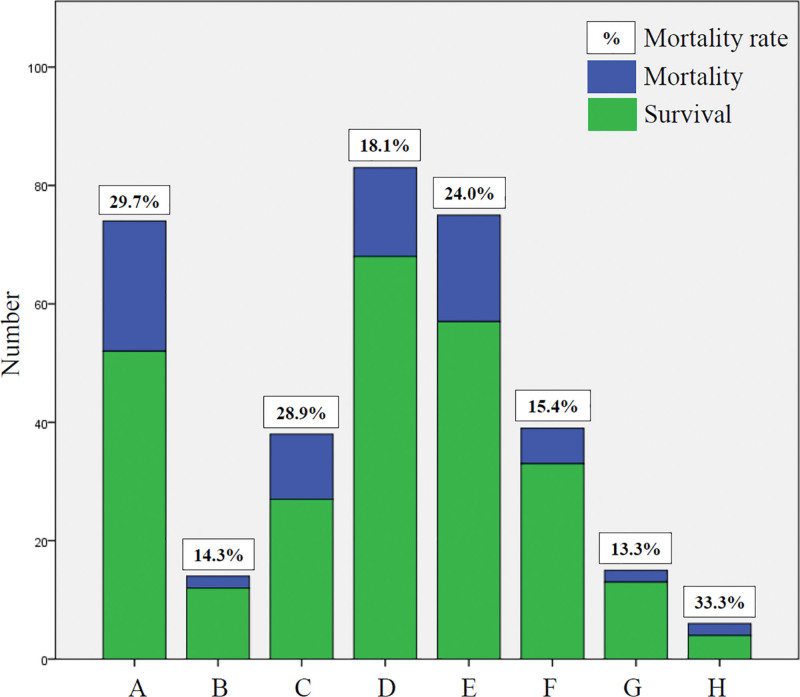
Analysis of in-hospital mortality in overall hospitalized COVID-19 patients with different dose (B–D) or type (E–H) of vaccines [(A) no vaccination (n = 74); (B) one dose of primary series (n = 14); (C) 2 doses of primary series (n = 38); (D) booster or additional dose (n = 83); (E) ChAdOx1-S-based primary series (n = 75); (F) mRNA-based (n = 39); (G) MVC-COV1901-based (n = 15); (H) mixing vaccines (n = 6)]. COVID-19 = coronavirus disease 2019.

The flow chart of patients screening was revealed on Figure 1. From May 2022 to July 2022, overall 213 COVID-19 patients without matching the criteria for release from isolation of Taiwan Centers for Disease Control, were admitted to the dedicated ward. Four cases with incomplete laboratory date were excluded from the statistical analysis. There were 209 cases enrolled in the statistical analysis, including 135 patients with COVID-19 vaccination and 74 patients without vaccination. Characteristics and outcomes were shown in Table 1. The analysis in different dose or type of vaccines was revealed on Figure 2 which demonstrated that the in-hospital mortality rate in overall patients only receiving primary series, were 14.3% with one dose of vaccine and 28.9% with 2 doses of vaccine. The mortality showed no statistical significance between one and 2 doses of primary series only (*P* = .472 by Fisher exact test) and the phenomenon might be attributed to the small sample size. The mortality rate in patients with booster or additional dose was 18.1%. The mortality rate were 24.0%, 15.4%, 13.3%, and 33.3% in those with basic primary series of ChAdOx1-S, mRNA vaccines, MVC-COV1901, and mixing vaccines.

**Table 1:** The characteristics and outcomes of COVID-19 hospitalized patients between vaccination and non-vaccination group.

	All n = 209	Vaccination n = 135	Non-vaccination n = 74	*P* value
Age; yr (mean)	74.7	74.6	75.0	.862
Male; n (%)	114 (54.5%)	70 (51.9%)	44 (59.5%)	.291
CCI (mean)	3.35	3.08	3.84	.034*
qSOFA; n (%)				
0	82 (39.2%)	60 (44.4%)	22 (29.7%)	
1	66 (31.6%)	42 (31.1%)	24 (32.4%)	
2	53 (25.4%)	28 (20.7%)	25 (33.8%)	
3	8 (3.8%)	5 (3.7%)	3 (4.1%)	
ALC under 800/μL; n (%)	79 (37.8%)	43 (31.9%)	36 (48.6%)	.017*
Anti-viral agents				
Oral agents; n (%)	66 (31.6%)	43 (31.9%)	23 (31.1%)	
Nirmatrelvir + ritonavir; n (%)	63 (30.1%)	40 (29.6%)	23 (31.1%)	
Molnupiravir; n (%)	3 (1.4%)	3 (2.2%)	0 (0%)	
3-day Remdesivir; n (%)	47 (22.5%)	29 (21.5%)	18 (24.3%)	
5-day Remdesivir; n (%)	81 (38.8%)	50 (37.0%)	31 (41.9%)	
Dexamethasone; n (%)	41 (19.6%)	25 (18.5%)	16 (21.6%)	.589
Mortality; n (%)	50 (23.9%)	28 (20.7%)	22 (29.7%)	.145
Hospital LOS; d (mean)	7.06	6.96	7.23	.878
Dedicated ward LOS; d (mean)	5.74	5.32	6.51	.080

ALC = absolute lymphocyte count, CCI = Charlson Comorbidity Index, LOS = length of stay, qSOFA = quick Sequential Organ Failure Assessment score.

* *P* < .05.

### 3.2. Clinical outcomes

**Figure 3.**
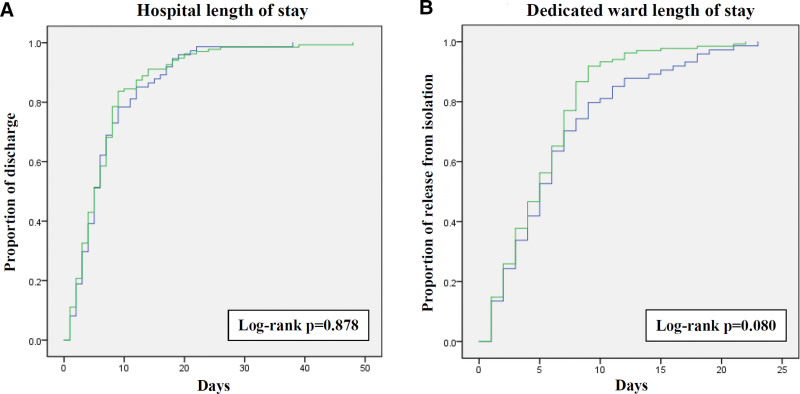
Hospital length of stay (A) and dedicated ward length of stay (B) curves of hospitalized COVID-19 patients with vaccination (green line) and without vaccination (blue line). COVID-19 = coronavirus disease 2019.

The overall in-hospital mortality rate was 23.9%. The mean of hospital LOS was 7.06 days and mean of dedicated ward LOS was 5.74 days. The in-hospital mortality rate were 20.7% and 29.7% in vaccination and non-vaccination group (*P* = .145). No significant difference was assumed in hospital LOS (*P* = .878) and somewhat shorter dedicated ward LOS was noted in vaccination group with marginal statistical significance (*P* = .080). The LOS curve plotted by Kaplan–Meier method was shown on Figure 3.

Multivariable analysis for in-hospital mortality rate by logistic regression which was shown on Table 2, identified age, CCI, qSOFA score and anti-virus agent as independent and strong prognostic indicators. As the age increased, the odds of in-hospital mortality also increased (aOR: 1.045; 95% CI: 1.011–1.080; *P* = .010). Increasing the CCI by one point was associated with a 1.2-fold increase in the odds of mortality (aOR: 3.730; 95% CI: 1.119–12.436; *P* = .032), and each additional point increase in the qSOFA was linked to a 2.7 times higher likelihood of mortality (aOR: 2.745; 95% CI: 1.537–4.905; *P* = .001). Patients with administration of 5-day course of remdesivir had 2.8 times higher odds of death than patients with other or without anti-viral agent (aOR: 2.847; 95% CI: 1.033–7.848; *P* = .043). After adjusting for multiple variables in the multivariable analysis, the effect of COVID-19 vaccination on the mortality became less significant. In subgroup analysis which was revealed on Table 3, vaccination demonstrated a significant reduction in the odds ratio of in-hospital mortality among the relatively young population.

**Table 2:** The multivariable logistic regression analysis for in-hospital mortality.

	cOR (95% CI)	*P* value	aOR (95% CI)	*P* value
Vaccination (ref = no)	0.619 (0.323–1.184)	.147	0.760 (0.328–1.761)	.522
Age	1.051 (1.021–1.081)	.001*	1.045 (1.011–1.080)	.010*
Gender (ref = female)	1.080 (0.570–2.048)	.813	1.052 (0.443–2.496)	.909
CCI	1.399 (1.216–1.611)	<.001*	1.215 (1.017–1.452)	.032*
qSOFA	4.449 (2.764–7.160)	<.001*	2.745 (1.537–4.905)	0.001*
ALC (ref = ≥800/μL)	0.904 (0.467–1.748)	0.764	0.580 (0.248–1.360)	0.210
Anti-viral agent (ref = except 5-day remdesivir)	8.543 (4.087–17.857)	<.001*	2.847 (1.033–7.848)	0.043*
Dexamethasone (ref = no)	5.789 (2.774–12.084)	<.001*	1.420 (0.490–4.118)	0.519

aOR = adjusted odds ratio, CCI = Charlson Comorbidity Index, CI = confidence interval, cOR = crude odds ratio, qSOFA = quick Sequential Organ Failure Assessment score, ref = reference.

* *P* < .05.

**Table 3:** The subgroup logistic regression analysis of vaccination for in-hospital mortality, stratified and adjusted by age, gender, Charlson Comorbidity Index, quick Sequential Organ Failure Assessment score, and absolute lymphocyte count group.

Vaccination (ref = no)	cOR (95% CI)	*P* value	aOR (95% CI)	*P* value
Age < 75; n = 84	0.208 (0.048–0.905)	.036*	0.094 (0.010–0.909)	.041*
Age ≥ 75; n = 125	0.868 (0.402–1.875)	.719	1.061 (0.436–2.583)	.896
Male; n = 114	0.536 (0.226–1.270)	.157	0.865 (0.283–2.639)	.799
Female; n = 95	0.755 (0.277–2.057)	.582	0.782 (0.244–2.505)	.678
CCI < 3; n = 89	0.386 (0.102–1.465)	0.162	0.348 (0.079–1.538)	.164
CCI ≥ 3; n = 120	0.812 (0.375–1.759)	0.597	1.123 (0.426–2.963)	.814
qSOFA < 2; n = 148	1.093 (0.362–3.307)	0.874	0.983 (0.297–3.251)	.997
qSOFA ≥ 2; n = 61	0.609 (0.220–1.690)	.341	0.645 (0.209–1.994)	.447
ALC ≥ 800/μL; n = 130	0.417 (0.181–0.963)	.040*	0.542 (0.179–1.638)	.278
ALC < 800/μL; n = 79	1.061 (0.368–3.053)	.913	1.222 (0.383–3.897)	.735

ALC = absolute lymphocyte count, aOR = adjusted odds ratio, CCI = Charlson Comorbidity Index, CI = confidence interval, cOR = crude odds ratio, qSOFA = quick Sequential Organ Failure Assessment score, ref = reference.

* *P* < .05.

## 4. Discussion

Comprehensive investigation of SARS-CoV-2 in recent years promoted the development of COVID-19 vaccines. The primary antigenic target for vaccines was the large surface spike protein, which bound to the angiotensin-converting enzyme 2 receptor on host cells. These COVID-19 vaccines were based on different technologies such as adenovirus vector vaccines [ChAdOx1-S (Oxford/AstraZeneca) and Ad26.COV2.S (Johnson & Johnson–Janssen)], messenger RNA (mRNA)-based vaccines [BNT162b2 (Pfizer–BioNTech) and mRNA-1273 (Moderna)], and adjuvanted recombinant protein vaccines [NVX-CoV2373 (Novavax) and MVC-COV1901 (Medigen)]. Previous studies revealed the efficacy and effectiveness of COVID-19 vaccination, with reduction of infection rate, disease severity, hospitalization and mortality.^[3–6]^

However, adaptive mutations in the genome of SARS-CoV-2 could alter the infectious potential. Since the beginning of the pandemic, there were 5 variants of concern containing Alpha (B.1.1.7), Beta (B.1.351), Gamma (P.1), Delta (B.1.617.2), and Omicron (B.1.1.529). The Omicron variant including many sub-variants, such as BA.1, BA.2, BA.3, BA.4, BA.5, BF.7, BQ.1.1, XBB, and descendent lineages, became the dominant strain in 2022 worldwide.

The immune escape of Omicron variant was described in recent studies.^[7,11,12]^ The ability to evade the immune protection elicited by previous infection or existing COVID-19 vaccines would create the great challenge of vaccine effectiveness. Andrews et al prescribed that primary immunization with 2 doses of vaccine provided limited protection against symptomatic disease caused by the Omicron variant.^[13]^ The booster or additional dose seemed indispensable in Omicron era. Even though the vaccine effectiveness of mRNA booster was short-lived in preventing infection, the protection against hospitalization and death was durable.^[13–17]^

The characteristics of overall population in our study leaned towards advanced age and a high prevalence of comorbidities. Despite less virulence of the Omicron variant compared to previous variants such as Delta, as discussed in the review by Naik et al,^[18]^ it continued to affect vulnerable populations in our study. Our results displayed that the in-hospital mortality rate was 20.7% in patients with vaccination and 29.7% in those without vaccination, without statistical significance. Somewhat shorter dedicated ward LOS was found in vaccination group with marginal statistical significance. In the vaccination group, a significantly lower average CCI and a reduced proportion of the population with an absolute lymphocyte count lower than 800 per microliter were observed, potentially impacting the outcomes. In addition, patients with multiple comorbidities might reduce the motivation to have COVID-19 vaccination. Vaccine hesitancy in individuals with serious comorbid conditions was reported and assumptions that the most vulnerable would automatically accept COVID-19 vaccination are erroneous.^[19]^

In multivariable analysis and subgroup analysis, we analyzed other prognostic factors affecting in-hospital mortality and found that the efficacy of the vaccination was influenced after adjustment. Other indicators dominated the clinical outcomes of COVID-19. Consistent with our study, age remained the strongest risk factor for severe COVID-19 outcomes, with risk of severe outcomes increasing markedly with increasing age.^[1]^ Vaccines demonstrated significant efficacy among relatively young populations in our subgroup analysis after adjustment. This observation could potentially imply that the vaccine’s efficacy might be comparatively diminished among individuals with advanced age. The risk factors of severe or critical COVID-19 should never be ignored. CCI was a universal tool to predict survival in patients with multiple comorbidities, despite the variation from the comorbidities classified as risk factors for severe COVID-19 by Centers for Disease Control and Prevention. Our multivariable analysis indicated CCI as a strong indicator for in-hospital mortality. Tuty Kuswardhani et al also reported that CCI score should be utilized for risk stratifications of hospitalized COVID-19 patients.^[20]^ qSOFA identified high-risk patients for in-hospital mortality with suspected infection. Citu et al^[21]^ reported that qSOFA was excellent predictor of in-hospital mortality among COVID-19 patients. Similar result was also found in our multivariable analysis. Some laboratory features associated with severe COVID-19 had been reported, including elevation in D-dimer, C-reactive protein, lactate dehydrogenase, troponin, ferritin, creatine-phospho-kinase, and decrease in absolute lymphocyte count.^[22–27]^ However in our study, the patients whose absolute lymphocyte count was lower than 800 per microliter did not had the higher odds of death.

The only anti-viral agent prescribed in severe COIVD-19 was 5-day course of remdesivir. The indication of other anti-viral agents including nirmatrelvir + ritonavir, molnupiravir and 3-day course of remdesivir, was mild disease with risk factors. It explained the higher odds of death in administration of 5-day remdesivir in our study. Corticosteroid was known as anti-inflammatory drug in severe COVID-19. Interestingly, dexamethasone prescription had higher odds of death in univariable analysis but the mortality had no significant difference multivariable analysis. It might imply the excellent efficacy of steroid or other main illness causing death.

Besides, the development of next-generation vaccine against Omicron variant might be expected in the future. Chalkias et al^[28]^ revealed that the bivalent Omicron-containing vaccine mRNA-1273.214 elicited neutralizing antibody responses against Omicron which were superior to those with mRNA-1273.

Our study had several limitations. First, this was a retrospective, single-center study which may infer selection bias and mis-classification or information bias. Second, the effectiveness of vaccination could not be evaluated due to lack of community-based study population, such as uninfected people and mild-disease patients with in-home care. Third, some patients with other principal diagnosis were admitted to the dedicated ward because of mismatching the criteria for release from isolation. It meant that COVID-19 might be not their main illness. Fourth, this district hospital in southern Taiwan did not set up the dedicated intensive care unit. Most COIVD-19 patients with severe or critical disease had signed Do-Not-Resuscitate (DNR) consent. Those who agreed with aggressive invasive medical treatment or procedure would be transferred to other evacuation hospitals. Finally, general vaccination needed about 2 weeks to reach the vaccine effectiveness.^[29–31]^ Some cases of our study who were admitted just after vaccination, might had inadequate time to build up the immunity.

In conclusion, the strong indicators including age, CCI and qSOFA, dominated the clinical outcomes of COVID-19. The current COVID-19 vaccines had significant efficacy in hospitalized COVID-19 patients in the relatively young group. The effect of vaccination may be relatively limited among hospitalized individuals of advanced age and multiple comorbidities.

## Acknowledgments

The authors would like to thank all colleagues who contributed to this study. The materials presented in this study were provided by Medical Expenditure Application Unit of Chi-Mei Medical Center, Chiali. The authors are grateful for the permission to use the materials.

## Author contributions

**Conceptualization:** Ming-Hung Chang.

**Data curation:** Ming-Hung Chang.

**Formal analysis:** Ming-Hung Chang.

**Investigation:** Ming-Hung Chang.

**Methodology:** Ming-Hung Chang.

**Project administration:** Ming-Hung Chang.

**Supervision:** Kuang-Ming Liao.

**Writing – original draft:** Ming-Hung Chang.

**Writing – review & editing:** Ming-Hung Chang, Kuang-Ming Liao.
